# Reuse of structural domain–domain interactions in protein networks

**DOI:** 10.1186/1471-2105-8-259

**Published:** 2007-07-18

**Authors:** Benjamin Schuster-Böckler, Alex Bateman

**Affiliations:** 1 Wellcome Trust Sanger Institute, Wellcome Trust Genome Campus, Hinxton, UK

## Abstract

**Background:**

Protein interactions are thought to be largely mediated by interactions between structural domains. Databases such as *i*Pfam relate interactions in protein structures to known domain families. Here, we investigate how the domain interactions from the *i*Pfam database are distributed in protein interactions taken from the HPRD, MPact, BioGRID, DIP and IntAct databases.

**Results:**

We find that known structural domain interactions can only explain a subset of 4–19% of the available protein interactions, nevertheless this fraction is still significantly bigger than expected by chance. There is a correlation between the frequency of a domain interaction and the connectivity of the proteins it occurs in. Furthermore, a large proportion of protein interactions can be attributed to a small number of domain interactions. We conclude that many, but not all, domain interactions constitute reusable modules of molecular recognition. A substantial proportion of domain interactions are conserved between *E. coli*, *S. cerevisiae *and *H. sapiens*. These domains are related to essential cellular functions, suggesting that many domain interactions were already present in the last universal common ancestor.

**Conclusion:**

Our results support the concept of domain interactions as reusable, conserved building blocks of protein interactions, but also highlight the limitations currently imposed by the small number of available protein structures.

## Background

One way to understand a protein's function is to look at its composition of conserved domains. Such families of related sequence regions, collected in the Pfam database [1], usually constitute structurally and functionally conserved modules. It is assumed that binding interfaces, too, are conserved evolutionary modules that are reused between proteins of different functions and retained during evolution [2,3].

Therefore, domain–domain interactions are often regarded as the currency of protein–protein interactions. Based on this assumption, Ng *et al*. described an approach to predict domain–domain interactions using literature curation, evolutionary history and the distribution of domains in protein interactions [4]. Wuchty *et al*. compared the relationship between this set of predicted interacting domain pairs to the domain coocurrence network [5]. More recently, other groups have come up with sophisticated statistical methods to estimate putatively interacting domain pairs, based on the assumption of domain reusability [6-10]. However, none of these approaches offers structural evidence that the predicted domain pairs are able to form an interaction.

For complexes with known structure, it has been shown that domains can mediate interactions [11,12]. Such interactions between pairs of domains are stored in the *i*Pfam database [13]. The structural evidence lends strong support to the inferred domain pair, resulting in a high confidence set of domain pairs.

Unfortunately, the selection of complexes in the PDB database of protein structures [14] is rather small and biased [15]. There is often only a single structure that shows a certain protein pair to interact, while other complexes like haemoglobin have been crystalized dozens of times. This makes it difficult to assess whether some domain pairs act as reusable modules in protein interactions from PDB data alone.

High-throughput experiments [16-18] and extensive literature curation efforts [19] have yielded large databases of protein interactions [20-24]. Despite the continuing growth of protein interaction databases, even the best studied protein interaction network of *S. cerevisiae *is thought to be incomplete and inaccurate [25-27]. Given that this network already comprises around 60000 interactions, questions arise as to how such networks have evolved and how they are organised. Furthermore, methods for assessing the quality of high-throughput experimental results are in high demand due to the error prone nature of the methods used.

In this study, we investigate how pairs of protein families taken from *i*Pfam are distributed in experimental protein interactions from five major model species. This allows us to address a number of questions: what proportion of each organism's protein interaction network, its *interactome*, can be attributed to a known domain–domain interaction? How conserved are domain–domain pairs between species, and how many interacting domain pairs are still unknown?

## Results

### iPfam domain pairs are overrepresented in experimental protein interactions

We analysed the distribution of Pfam families known to interact from a PDB structure (*iPfam domain pairs*) in experimentally derived protein interactions (*experimental interactions*). The experimental interactions were filtered to only include interactions with exactly two partners (see Methods). The fraction of experimental interactions that contain at least one *i*Pfam domain pair is referred to as the *iPfam coverage*. Accordingly, the fraction of experimental interactions that contains any pair of Pfam domains (excluding the *i*Pfam domain pairs) is called the *Pfam coverage*.

**Figure 1:**
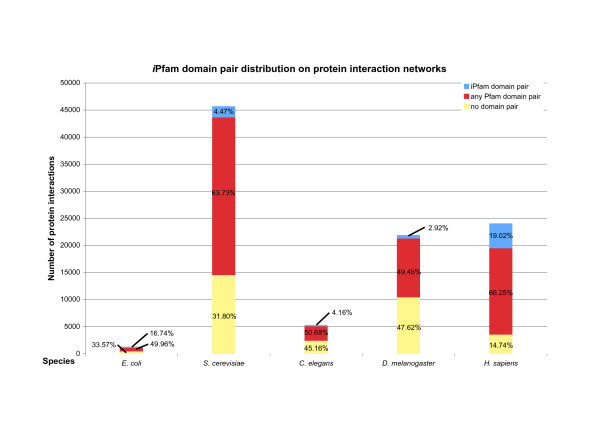
**Comparison of coverage of iPfam domain pairs on protein interactions**. For each species, the height of the column reflects the number of known protein–protein interactions in the data set. The columns are split according to the proportion of interactions that contain an *i*Pfam domain pair (blue), that contain any other Pfam domains on both proteins (red), and those that contain no Pfam domain pair (yellow).

Figure 1 shows the Pfam and *i*Pfam coverage for the analysed species as a column chart. The number of resolved protein interactions varies greatly between species, as does the size of the underlying proteome (see Table 1). The Pfam coverage, coloured red in Figure 1, lies between 49.46% and 66.73%. Given that 74% of all UniProt proteins contain at least one Pfam match, this is not by itself surprising. The *i*Pfam coverage, shown in blue, is much smaller, ranging from 2.92% in *D. melanogaster *to 19.02% in *H. sapiens*. In *S. cerevisiae *the species with the most comprehensively studied interactome, the *i*Pfam coverage is 4.47%.

**Table 1:** iPfam domain pair coverage on protein interactions

**Species**	**Proteins in proteome**	**% proteome in interaction set**	**Protein pairs in interaction set**	**Protein pairs with iPfam domain pair**	**Protein pairs with iPfam domain pair (randomised mean)**	**Standard deviation**
*E. coli*	4314	26.96%	1281	211	178	7.12
*S. cerevisiae*	5780	92.72%	45707	2045	528	57.49
*C. elegans*	22437	13.47%	5310	221	76	9.90
*D. melanogaster*	16251	43.22%	21921	641	195	21.79
*H. sapiens*	38213	21.40%	24065	4577	1373	116.86

For each species, we list the size of the proteome as defined in Integr8 and the fraction of this proteome that is represented in the protein interaction sets, followed by the total number of binary protein interactions and the fraction of those that contain an *i*Pfam domain pair. The last two columns show the results of the network shuffling experiments.

The relatively low *i*Pfam coverage is by itself a disappointing finding. However, the fact that only a small fraction of protein interactions contain known domain pairs could be a result of the scarcity of available structures of protein complexes. Therefore, we asked whether the observed *i*Pfam coverage is larger than would be expected by chance. To test this, we created 1000 random networks per species using the algorithm described in Methods. We then calculated the *i*Pfam coverage on the protein interactions in each randomised network. Mean and standard deviations of the randomisation experiments are shown in Table 1. No P value (see Methods) was greater than 1.84 · 10^-06^. This proves that the observed *i*Pfam coverage is significantly higher than expected and *i*Pfam domain pairs are enriched in real experimental protein interactions.

### Few iPfam domain pairs are responsible for a majority of the coverage

**Figure 2:**
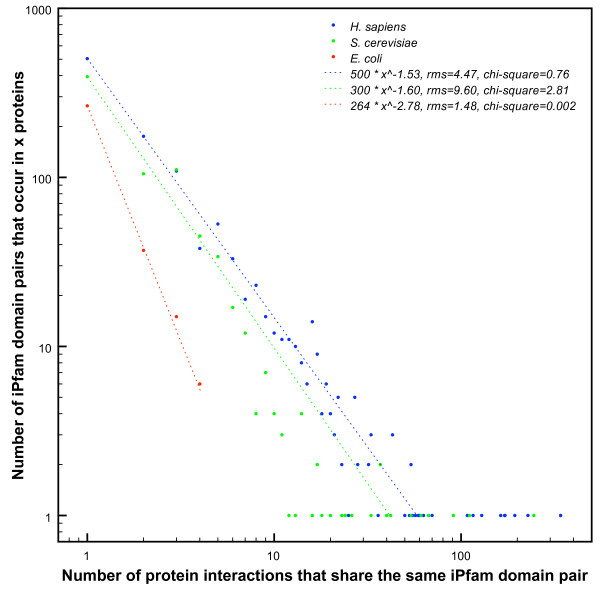
**Frequencies of iPfam domain pairs in E. coli, S. cerevisiae and H. sapiens protein interactions**. Each point in this graph represents a set of protein interactions. The abscissa reflects the number of interactions in each set that contain the same *i*Pfam domain pair. The ordinate shows the number of distinct such sets, each defined by a different *i*Pfam domain pair. In both *H. sapiens *(blue) and *S. cerevisiae *(green) a small number of *i*Pfam domain pairs covers a large fraction of the interactome, whereas in *E. coli*, no *i*Pfam domain occurs in more than 4 experimental interactions at a time. Dotted lines denote fitted monomial functions, showing that the distributions follow a power law.

To understand why *i*Pfam domain pairs occur more often in experimental interactions than expected by chance, we analysed the two largest data sets, *S. cerevisiae *and *H. sapiens *in more detail. In the following paragraph, we will call the experimental interactions that contain an *i*Pfam domain pair the *covered experimental interactions*. In Figure 2, we compare the distribution of *i*Pfam domain pairs on the number of experimental interactions for *E. coli*, *S. cerevisiae *and *H. sapiens*. This plot reflects how many *i*Pfam domain pairs cover how many experimental interactions. Domain pairs that cluster to the left of the plot can be called *specific *domain pairs, as they only occur in very few covered experimental interactions. Conversely, domain pairs that cluster to the right of the plot occur in a large number of different covered experimental interactions and can be called *promiscuous *domain pairs.

All three distributions in Figure 2 resemble a power law distribution, according to the good fit of log-linear functions (log(*f*(*x*)) = *k *log *x *+ log *a*) shown as dotted lines. The slopes *k *of the *H. sapiens *and *S. cerevisiae *distributions are very similar (-1.53 and -1.60, respectively), while *E. coli *has a markedly smaller slope (-2.78). This suggests that the ratio of specific to promiscuous *i*Pfam domain pairs is very similar in *S. cerevisiae *and *H. sapiens*, whereas *E. coli *features fewer multiply reoccurring *i*Pfam domain pairs.

The power law distribution of *i*Pfam frequencies implies that the majority of covered protein interactions can be attributed to a minority of *i*Pfam domain pairs. 51.7% of the *i*Pfam domain pairs in *S. cerevisiae *and 45.3% in *H. sapiens *are seen in just one experimental interaction. Conversely, 92.4% of *H. sapiens *and 85.4% of *S. cerevisiae *covered experimental interactions contain an *i*Pfam domain pair that occurs more than once. Even more, half of the covered experimental interactions in *H. sapiens *contain an *i*Pfam domain pair that occurs in more than 16 different experimental interactions (5 for *S. cerevisiae*).

### Degree distribution and iPfam domain pair frequency are correlated

We reasoned that if there are *i*Pfam domain pairs that act as reusable modules in protein interactions, then highly connected proteins should also be more likely to contain promiscuous *i*Pfam domain pairs and vice-versa.

**Figure 3:**
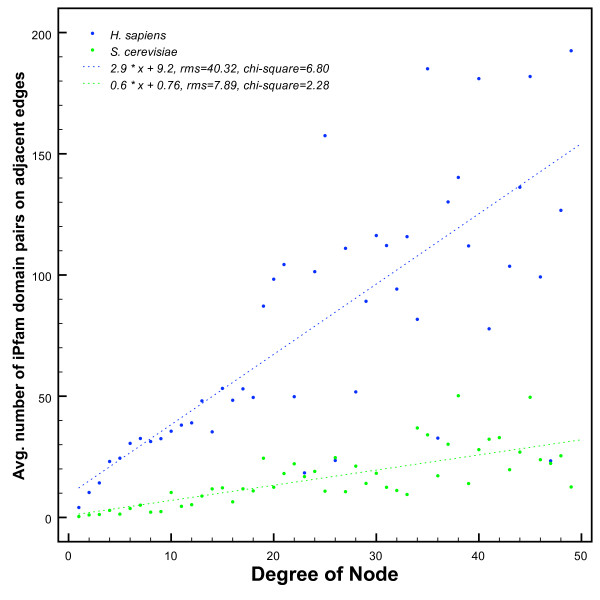
**Average frequency of iPfam domain pairs relative to degree of node**. Each point represents a protein in the interaction networks of *H. sapiens *(blue) and *S. cerevisiae *(green). For each protein, we calculate the degree, defined as the number of interactions the protein is involved in. On the y-axis, we show the average number of *i*Pfam domain pairs in edges adjacent to proteins of degree *x*. We calculated a Spearman correlation of 0.68 and 0.71, for *H. sapiens *and *S. cerevisiae*. The correlation is outlined by dotted lines.

For each node (i.e. protein) in the filtered *H. sapiens *and *S. cerevisiae *protein interaction network, we calculated its degree, defined as the number of adjacent edges (i.e. interactions). At the same time, we counted the number of *i*Pfam domain pairs on the adjacent edges. In Figure 3, we plot the mean number of *i*Pfam domain pairs relative to the degree of the node.

We find that for proteins from a degree of 1 to 50, there is strong correlation in both *H. sapiens *and *S. cerevisiae *(Spearman correlation coefficients of 0.68 and 0.71, respectively) between degree and number of *i*Pfam domain pairs on adjacent edges. For the 1.2% of proteins in *H. sapiens *and 6.4% in *S. cerevisiae *which have a degree higher than 50, the correlation gradually diminishes.

### Promiscuous domain pairs

Additional file 1 contains a list of all *i*Pfam domain pairs and their frequencies in the experimental protein interactions, while Additional file 4 lists the frequencies of the single domains. Interactions between protein kinase domains (Pkinase, Pfam acc. PF00069 and Pkinase_Tyr, Pfam acc. PF07714) are the most frequent *i*Pfam domain pairs, as well as interactions involving recognition domains such as SH2 or SH3. In *S. cerevisiae*, the Proteasome family (Pfam acc. PF00227, a family of peptidases) and WD40 (Pfam acc. PF00400, a repeat involved in multimer assembly) are also amongst the five most frequent *i*Pfam domain pairs. As expected, more frequent domains are also more likely to be found as pairs in interacting proteins. It should be noted however that in the PDB structures, some of the observed domain pairs (Pkinase_Tyr ↔ SH3_1, Pkinase_C ↔ Pkinase and others) are only seen to interact within one protein (intrachain interactions) as opposed to interactions between two distinct proteins (interchain interaction). The table in Additional file 5 lists the number of PDB structures for each *i*Pfam domain pair, distinguishing between intrachain and interchain interactions. Looking for example at the covered experimental interactions in *H. sapiens*(Additional file 1), only 8 out of the 100 most frequent *i*Pfam domain pairs are seen in intrachain interactions exclusively, while 61 are exclusive to interchain interactions and 31 are seen in both.

A possible explanation for the occurrence of purely intrachain *i*Pfam domain pairs in the covered experimental interactions is that they frequently cooccur together on the same protein with other *i*Pfam domain pairs. A list of all combinations of *i*Pfam domains (the *domain architecture*) on interacting proteins is given in Additional file 2. It reveals that certain *i*Pfam domains such as SH2, SH3_1 or Pkinase_tyr frequently occur in the same architecture. Without further experiments, we cannot assign the correct interacting domains with certainty.

This highlights a basic assumption of this study that could be a source of error. We assume that interacting proteins that contain an *i*Pfam domain pair interact through these domains. This, of course, is not necessarily the case. Although it has been shown that sequence similarity is linked to the mode of interaction [28], not every protein interaction that contains an *i*Pfam domain pair is necessarily mediated by exactly this domain pair. To gain a rough estimate of the false positive rate due to this assumption, we counted how many protein pairs in the PDB contain an *i*Pfam domain pair that does not mediate an interaction in one complex structure but does so in another. 3671 out of a total of 5380 interacting protein pairs from the PDB contain an *i*Pfam domain pair that does not interact in one complex structure but does so in another. This means that for more than 32% of the protein interactions in the PDB, the *i*Pfam domain pair assignment is correct. For the remaining 68%, the *i*Pfam domain pair assignments are wrong in one case but correct in another. The real false positive rate is likely to be smaller, because some *i*Pfam domain pairs might still independently mediate an interaction with a different, possibly unknown, partner protein.

### iPfam domain pairs are enriched in S. cerevisiae complexes

We tested whether *i*Pfam domain pairs are enriched in known protein complexes from *S. cerevisiae*. This is interesting firstly because domain–domain interactions are thought to be more common in obligate interactions. Secondly, the described modularity of known *S. cerevisiae *complexes lends support to the assumption that the underlying *i*Pfam domain pairs are modular. In fact, we find a two-fold enrichment for *i*Pfam domain pairs in the complexes described by Gavin *et al*. [29]. From the 294 binary protein interactions in this data set, 24 contained an *i*Pfam domain pair, which corresponds to a coverage of 8.16% (P value 2.7 · 10^-47^).

We also analysed the full dataset of protein complexes. From 491 complexes described by Gavin *et al*., 157 contained at least one pair of proteins with an *i*Pfam domain pair (31.9%). In total we found 617 pairs of proteins that contained an *i*Pfam domain pair. Interestingly, we find that the distribution of *i*Pfam domain pairs on complexes is uneven. When we drew 617 protein pairs randomly from all possible protein pairs in the complexes, we covered 192 complexes on average, with a standard deviation of 7.22. The probability of covering only 157 complexes is just 6.24 · 10^-07^. Thus, some complexes contain a greater number of *i*Pfam domain pairs, while other complexes do not contain any at all. This suggests that some sets of domain pairs are specific to certain complexes or pathways. Typical examples are the RNA polymerase II complex (IntAct id: EBI-815049) or the U1 snRNP complex which contain numerous *i*Pfam domain pairs that are specific to these complexes.

### iPfam domain pairs are conserved between species

Within the 3 to 19% of experimental interactions covered by *i*Pfam, we analysed the conservation of *i*Pfam domain pairs between species. We call an *i*Pfam domain pair *conserved *when the same pair is observed in experimental interactions of two different species. The matrix in Table 2 shows the pair-wise conservation of *i*Pfam domain pairs. For each species, a maximum of 40% to 90% of *i*Pfam domain pairs can also be found in another species, although not all overlaps are as large.

**Table 2:** Matrix of mutual shared iPfam domain pairs

	*E. coli*	*S. cerevisiae*	*C. elegans*	*D. melanogaster*	*H. sapiens*	*i*Pfam domain pairs in total
*E. coli*		158	35	30	135	347
*S. cerevisiae*			129	164	524	835
*C. elegans*				102	172	197
*D. melanogaster*					241	266
*H. sapiens*						1221

The Table shows the number of co-occurences of *i*Pfam domain pairs between two species. The right-most column lists the total number of unique *i*Pfam pairs found in each species' experimental interactions.

**Figure 4:**
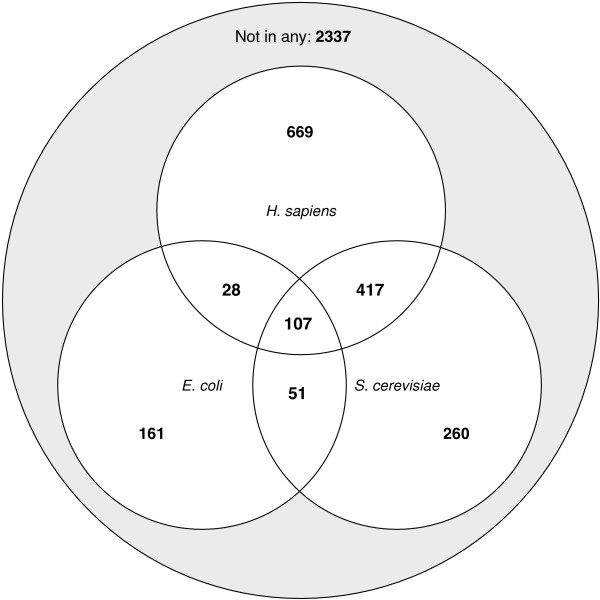
**Venn diagramm showing the fractions of iPfam domain pairs found in the E. coli, S. cerevisiae and H. sapiens binary protein interaction sets**. The three circles represent the *i*Pfam domain pairs observed in the respective species. The overlaps denote co-observed *i*Pfam domain pairs. The grey set in the background represents *i*Pfam domain pairs not found in the three species.

Figure 4 shows a Venn diagram of the mutual overlaps between the two eukaryotes *S. cerevisiae *and *H. sapiens *and the prokaryote *E. coli*. While the eukaryotes share 524 domain pairs, only 158 *i*Pfam domain pairs are shared between *S. cerevisiae *and *E. coli*, and only 135 between *E. coli *and *H. sapiens*. Remarkably, 53% of the observed *i*Pfam domain pairs in *E. coli *are also observed in one of the two eukaryotes, and 107 *i*Pfam domain pairs are even conserved amongst all three species. The *i*Pfam domains in these pairs are related to housekeeping activities such as translation, replication or ATP synthesis. Additional file 3 contains a list of the conserved *i*Pfam domain pairs.

We also compared the *i*Pfam domain pair frequencies between *H. sapiens *and *S. cerevisiae *directly. We derive a Spearman correlation coefficient of 0.50 for the frequencies of all 524 *i*Pfam domain pairs that are conserved between *S. cerevisiae *and *H. sapiens*. To test whether the correlation is an artefact of the distribution of the values, we recalculated the correlation 1000 times, each time shuffling one distribution randomly. From these random results, we derive a P value of 3.6 · 10^-30 ^that the observed correlation is random. This suggests that *i*Pfam domain pairs with a large number of occurrences in one species tend also to be more frequent in the other.

### Predicting the total number of iPfam domain pairs in nature

Our analysis allow us to estimate how many *i*Pfam domain pairs would eventually cover all protein interactions. This corresponds to the predictions made by Aloy and Russel [2]. Similar to their approach, we make a linear estimation with the following factors:

*χ*_*S *_The number of *i*Pfam domain pairs observed in species *S*

*θ*_*S *_The number of observed interactions in species *S *that contain an *i*Pfam domain pair

Θ_*S *_The total number of observed interactions in species *S*

*ψ*_*S *_The number of proteins from species *S *that are seen in an interaction screen

Ψ_*S *_The proteome size for species *S*

*ξ*_*S *_The number of Pfam domains observed in all protein of species *S*

Ξ The total number of known Pfam domains

We denote the estimated number of *i*Pfam domain pairs in species *S *with x^S
 MathType@MTEF@5@5@+=feaafiart1ev1aaatCvAUfKttLearuWrP9MDH5MBPbIqV92AaeXatLxBI9gBaebbnrfifHhDYfgasaacH8akY=wiFfYdH8Gipec8Eeeu0xXdbba9frFj0=OqFfea0dXdd9vqai=hGuQ8kuc9pgc9s8qqaq=dirpe0xb9q8qiLsFr0=vr0=vr0dc8meaabaqaciaacaGaaeqabaqabeGadaaakeaacuWG4baEgaqcamaaBaaaleaacqWGtbWuaeqaaaaa@2F90@. The formula we apply is

x^S=χS⋅ΘSθS⋅ΨSψS
 MathType@MTEF@5@5@+=feaafiart1ev1aaatCvAUfKttLearuWrP9MDH5MBPbIqV92AaeXatLxBI9gBaebbnrfifHhDYfgasaacH8akY=wiFfYdH8Gipec8Eeeu0xXdbba9frFj0=OqFfea0dXdd9vqai=hGuQ8kuc9pgc9s8qqaq=dirpe0xb9q8qiLsFr0=vr0=vr0dc8meaabaqaciaacaGaaeqabaqabeGadaaakeaacuWG4baEgaqcamaaBaaaleaacqWGtbWuaeqaaOGaeyypa0dcciGae83Xdm2aaSbaaSqaaiabdofatbqabaGccqGHflY1daWcaaqaaiabfI5arjabdofatbqaaiab=H7aXnaaBaaaleaacqWGtbWuaeqaaaaakiabgwSixpaalaaabaGaeuiQdK1aaSbaaSqaaiabdofatbqabaaakeaacqWFipqEdaWgaaWcbaGaem4uamfabeaaaaaaaa@444B@

This means we scale the observed number of *i*Pfam domain pairs to cover all observed interactions. We then use the relative proteome coverage to estimate the total number of *i*Pfam domain pairs in all proteins.

Finally, we follow the argument of Aloy and Russel that the number of Pfam families seen in species *S *indicates the fraction of the protein universe represented in the species. We therefore predict the total number of *i*Pfam domain pairs x^
 MathType@MTEF@5@5@+=feaafiart1ev1aaatCvAUfKttLearuWrP9MDH5MBPbIqV92AaeXatLxBI9gBaebbnrfifHhDYfgasaacH8akY=wiFfYdH8Gipec8Eeeu0xXdbba9frFj0=OqFfea0dXdd9vqai=hGuQ8kuc9pgc9s8qqaq=dirpe0xb9q8qiLsFr0=vr0=vr0dc8meaabaqaciaacaGaaeqabaqabeGadaaakeaacuWG4baEgaqcaaaa@2E35@ as

x^=x^S⋅ΞξS
 MathType@MTEF@5@5@+=feaafiart1ev1aaatCvAUfKttLearuWrP9MDH5MBPbIqV92AaeXatLxBI9gBaebbnrfifHhDYfgasaacH8akY=wiFfYdH8Gipec8Eeeu0xXdbba9frFj0=OqFfea0dXdd9vqai=hGuQ8kuc9pgc9s8qqaq=dirpe0xb9q8qiLsFr0=vr0=vr0dc8meaabaqaciaacaGaaeqabaqabeGadaaakeaacuWG4baEgaqcaiabg2da9iqbdIha4zaajaWaaSbaaSqaaiabdofatbqabaGccqGHflY1daWcaaqaaiabf65aybqaaGGaciab=57a4naaBaaaleaacqWGtbWuaeqaaaaaaaa@392C@

Both parameters and results of the calculation are shown in Table 3. The estimates for the total number of *i*Pfam domain pairs ranges from 33813 to 120511, with an average of 76918.

**Table 3:** Prediction of total number of iPfam domain pairs

Species	Θ_*S*_	*θ*_*S*_	Ψ_*S*_	*ψ*_*S*_	*χ*_*S*_	x^S MathType@MTEF@5@5@+=feaafiart1ev1aaatCvAUfKttLearuWrP9MDH5MBPbIqV92AaeXatLxBI9gBaebbnrfifHhDYfgasaacH8akY=wiFfYdH8Gipec8Eeeu0xXdbba9frFj0=OqFfea0dXdd9vqai=hGuQ8kuc9pgc9s8qqaq=dirpe0xb9q8qiLsFr0=vr0=vr0dc8meaabaqaciaacaGaaeqabaqabeGadaaakeaacuWG4baEgaqcamaaBaaaleaacqWGtbWuaeqaaaaa@2F90@	Ξ	*ξ*_*S*_	x^ MathType@MTEF@5@5@+=feaafiart1ev1aaatCvAUfKttLearuWrP9MDH5MBPbIqV92AaeXatLxBI9gBaebbnrfifHhDYfgasaacH8akY=wiFfYdH8Gipec8Eeeu0xXdbba9frFj0=OqFfea0dXdd9vqai=hGuQ8kuc9pgc9s8qqaq=dirpe0xb9q8qiLsFr0=vr0=vr0dc8meaabaqaciaacaGaaeqabaqabeGadaaakeaacuWG4baEgaqcaaaa@2E35@
*E. coli*	1281	211	4314	1163	347	**7814**	8957	2070	**33813**
*S. cerevisiae*	45707	2045	5780	5359	835	**20129**	8957	2119	**85085**
*C. elegans*	5310	221	22437	3022	197	**35143**	8957	2612	**120511**
*D. melanogaster*	21921	641	16251	7023	266	**21049**	8957	2777	**67893**
*H. sapiens*	24065	4577	38213	8179	1221	**29994**	8957	3476	**77288**

Θ_*S *_The total number of observed interactions in species *S*

*θ*_*S *_The number of observed interactions in species *S *that contain an *i*Pfam domain pair

Ψ_*S *_The proteome size for species *S*

*ψ*_*S *_The number of proteins from species *S *that are seen in an interaction screen

*χ*_*S *_The number of *i*Pfam domain pairs observed in species *S*

x^S
 MathType@MTEF@5@5@+=feaafiart1ev1aaatCvAUfKttLearuWrP9MDH5MBPbIqV92AaeXatLxBI9gBaebbnrfifHhDYfgasaacH8akY=wiFfYdH8Gipec8Eeeu0xXdbba9frFj0=OqFfea0dXdd9vqai=hGuQ8kuc9pgc9s8qqaq=dirpe0xb9q8qiLsFr0=vr0=vr0dc8meaabaqaciaacaGaaeqabaqabeGadaaakeaacuWG4baEgaqcamaaBaaaleaacqWGtbWuaeqaaaaa@2F90@ The predicted total number of *i*Pfam domain pairs in species *S*

Ξ The total number of known Pfam domains

*ζ*_*S *_The number of Pfam domains observed in all protein of species *S*

x^
 MathType@MTEF@5@5@+=feaafiart1ev1aaatCvAUfKttLearuWrP9MDH5MBPbIqV92AaeXatLxBI9gBaebbnrfifHhDYfgasaacH8akY=wiFfYdH8Gipec8Eeeu0xXdbba9frFj0=OqFfea0dXdd9vqai=hGuQ8kuc9pgc9s8qqaq=dirpe0xb9q8qiLsFr0=vr0=vr0dc8meaabaqaciaacaGaaeqabaqabeGadaaakeaacuWG4baEgaqcaaaa@2E35@ The estimated total number of *i*Pfam domains in all species

Prediction results are shown in bold font.

## Discussion

### iPfam coverage is low

The coverage of *i*Pfam on experimentally derived protein interactions is low. For *S. cerevisiae*, the species with the best mapped interactome, only 4.47% of the protein interactions contain an *i*Pfam domain pair. Even in *H. sapiens*, where we suspect a positive bias due to the overrepresentation of disease-related proteins in both the PDB and protein interaction databases, 81% of protein interactions do not contain an *i*Pfam domain pair. This reveals the limits of our understanding of the molecular structure of protein interactions.

Figure 1 also shows that a majority of protein interactions contains at least one pair of Pfam domains.  While there is no structural information about putative interactions between these pairs, this fraction can already be analysed using statistical methods to identify putative domain interactions [7,9,10]. This in turn creates new targets for future structural genomics projects [30]. Prioritising these targets according to the number of covered experimental interactions could increase the coverage of databases like *i*Pfam quickly.

We find, however, that *i*Pfam domain pairs occur significantly more often in experimental interactions than would be expected by chance. This requires that at least a subset of the *i*Pfam domain pairs are reused in several experimental interactions.

### iPfam domain pairs can act as modules

Despite the low overall coverage, *i*Pfam domain pairs are found in more protein interactions than would be expected by chance (see Table 1). This statistical overrepresentation suggests that certain *i*Pfam domain pairs constitute modules of molecular recognition which are reused in different protein interactions [2]. In fact, we find a characteristic power law distribution when we plot the histogram of experimental interactions per *i*Pfam domain pair, see Figure 2. This underlines that a few promiscuous *i*Pfam domain pairs are responsible for the majority of the *i*Pfam coverage. These *i*Pfam domain pairs are most likely to be reusable modules. In fact, we find the most frequent *i*Pfam domain pairs to be recognition domains in signal transduction. Conversely, a large number of *i*Pfam domain pairs are specific to a small number of protein interactions. This implies that recognition specificity amongst proteins is often achieved by maintaining an exclusive interacting domain pair. This could pose a problem for purely statistical approaches to infer domain interactions: if for many interfaces the real interacting domain pair will only occur once in an interactome, it will be hard to elucidate this on a statistical basis.

The concept of modularity of interacting domain pairs is furthermore supported by the positive correlation between the number of protein interactions an *i*Pfam domain pair is seen in and the connectivity of the interacting proteins. We hypothesise that if during the course of evolution a protein is duplicated, it is likely to retain connections with other proteins which contain the same domain interaction modules. It is clear, however, that even though recognition domains are reused in various proteins, their specificity is bound to be controlled.

### Many domain–domain interfaces remain to be resolved

We tried to estimate how many *i*Pfam domain pairs exists in all interactomes. Our predictions lie almost an order of magnitude higher than the 10000 domain interaction types proposed by Aloy and Russel [2]. While all such estimates should be taken with caution, our results show that at best 10% of all structural domain pairs are represented in *i*Pfam. The statistical approaches described in the introduction can only cover a small fraction of this domaininteraction space. Riley *et al*. for example report only 3005 interacting domain pairs which could be inferred from protein interactions [7]. Even under the assumption that many interactions involve short linear motifs, it seems likely that a large number of domain interactions remain to be resolved.

### iPfam domain pairs are conserved during evolution

*i*Pfam domain pairs are not only recurrent within the protein interaction network of one species. They also appear to be conserved between species. In a small set of protein structures from *S. cerevisiae*, it has been shown that interacting domain pairs are more conserved than non-interacting domain pairs [10]. Here, we call an *i*Pfam domain pair conserved if there are protein interactions in two species which contain the same *i*Pfam domain pair.

In a recent study [31], Gandhi *et al*. have assessed the conservation of protein interactions as the co-occurrence of orthologous interacting proteins. They found only 16 orthologous interacting protein pairs that were conserved in *S. cerevisiae*, *C. elegans*, *D. melanogaster *and *H. sapiens*. Conversely, we find that 71 *i*Pfam domain pairs are conserved in the experimental interactions of these species. Even between a prokaryote like *E. coli *and the two eukaryotes *S. cerevisiae *and *H. sapiens *there is a considerable proportion of conserved *i*Pfam domain pairs, to the extent that 53% of the *i*Pfam domain pairs from *E. coli *are also observed in a eukaryote (Table 2). 107 domain pairs are shared between *E. coli*, *S. cerevisiae *and *H. sapiens*. These domains are predominantly related to transcription, translation and other basic essential cellular activities, which is in congruence with the findings of Gandhi *et al*..

Although the low overall *i*Pfam coverage hampers the interpretation of our results, it looks as if there has been a diversification of domain interactions from *E. coli *to *H. sapiens*. While more than half of the *i*Pfam domain pairs in *E. coli *have been retained throughout evolution, numerous new ones seem to have emerged in eukaryotic development. The significant positive correlation in the frequency of *i*Pfam domain pairs conserved between *S. cerevisiae *and *H. sapiens *also suggests that the binding interfaces are more often kept or even reused rather than lost in the course of evolution. Conversely, this also raises the question of whether one could establish a comprehensive set of domain interactions that were present in the last universal common ancestor.

## Conclusion

In this study, we addressed the utility of current knowledge about structural domain interactions in order to interpret experimental protein interactions. Disappointingly, only a small fraction of all experimental interactions can be attributed to a known domain interaction. Within this subset of interactions, we nevertheless made several reassuring observations: structural domain pairs are enriched in experimental protein interactions. Some of the domain pairs seem to mediate a large number of protein interactions, thus acting as reusable connectors. This property is also conserved between species. Taken as a whole, this further underlines that solving structures of protein complexes should be an important focus for future structural genomics projects. Targeting the most frequent domain pairs would increase the coverage of databases such as *i*Pfam, shedding more light onto the molecular mechanisms underpinning cellular networks.

## Methods

### Protein interaction data

The complete interaction sets from BioGRID [20], DIP [21], HPRD [22], IntAct [23] and MPact [24] were downloaded. A wide range of databases were used to cover as many distinct experimental data sets as possible. BioGRID for example contains a large manually curated set of protein interactions for *S. cerevisiae *[19]. Similarily, HPRD hosts a set of manually curated protein interactions for *H. sapiens*. IntAct on the other hand contains results from high-throughput screens and integrates data from other protein interaction databases as part of the IMEx collaboration. The MPact database combines the manually curated *S. cerevisiae *protein complexes data set formerly known as the MIPS complexes with other high-throughput interaction experiments data. Taken together, these databases represent most of the protein interactions currently stored in machine-accessible form.

Despite great efforts to unify access to protein interaction data [32], acquiring large data sets from diverse sources is still far from trivial and error prone. The PSI-MI XML data exchange format provided by the aforementioned databases was used to generate a local relational database of protein interactions. All entries were mapped to UniProt [33] by either relying on existing annotations from the source databases or by pair-wise sequence alignment to all UniProt proteins from the same species as the query protein. The direct sequence comparison was performed using pmatch, a very fast pairwise alignment algorithm developed by Richard Durbin (unpublished, source code available [34]).

### Species

To allow cross-species comparisons, the data were split into five distinct species sets: *E. coli*, *S. cerevisiae*, *C. elegans*, *D. melanogaster *and *H. sapiens*. It should be noted that the proportion of proteins for which an interaction is known varies greatly between the species, see Table 1. This might affect the results if there is a systematic bias on the composition of a protein interaction set.

To prevent bias from multiple alternative versions of the same protein, all interacting proteins were mapped to reference proteomes as defined by Integr8 [35], again using pmatch. An average of ≈ 16% of interaction entries were lost in the mapping process, either if no sequence was provided with the original entry or if no significant matching sequence could be found in Integr8. The total number of missing proteins will be lower, as several entries from different databases refer to the same sequence.

### iPfam

**Figure 5:**
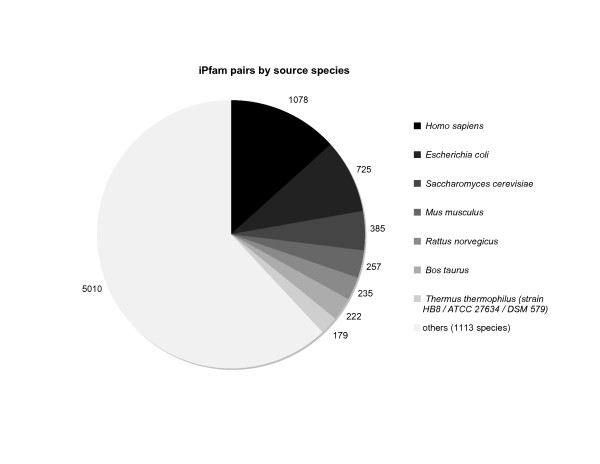
**Species distribution of iPfam domain pairs**. This pie chart shows how many *i*Pfam domain pairs were found in PDB structures from each species. The total number is larger than the 4030 unique *i*Pfam pairs in the database because an *i*Pfam pair can be found in structures from several species.

The *i*Pfam database is derived from protein structures deposited in the PDB. Regions in every protein structure that match a Pfam domain are scanned for interactions with residues in another Pfam domain. All such interacting domain pairs are stored in a database together with detailed information on the residues involved [13]. Every *pair *of Pfam families that are found to interact in a PDB structure are called an *iPfam domain pair *throughout the text. Single Pfam families that are part of an *i*Pfam domain pair are then called *iPfam domains*. For example, in PDB entry 1k9a the two *i*Pfam domains SH2 (Pfam accession PF00017) and Pkinase_Tyr (PF07714) interact, therefore they form an *i*Pfam domain pair. In this study, *i*Pfam version 21 was employed, containing 2837 *i*Pfam domains, forming 4030 *i*Pfam domain pairs. Figure 5 shows the species distribution of *i*Pfam domain pairs. *H. sapiens*, *E. coli *and *S. cerevisiae *are clearly over-represented compared to the other 1113 species with less than 179 complex structures. Some *i*Pfam domain pairs are seen to form interactions between distinct peptide chains in the structure (*interchain*), while others form an interaction between two distinct domains within the same chain (*intrachain*). In *i*Pfam version 21, there are 3407 interchain and 1171 intrachain domain pairs, which means that 548 domain pairs mediate both inter- and intrachain interactions. In this analysis, both types of domain interactions were used equivalently, assuming that intrachain interactions can become interchain interactions and *vice-versa *as a result of a gene-fission/fusion events.

### Filtering

There are many types of experiments used to derive protein interactions, with different properties and error rates. For this analysis, solely the properties of physically interacting proteins is of interest. Therefore, only interactions between exactly two proteins per experiment were considered. That means all protein complex data that were derived by co-purification methods were removed, unless a particular experiment had identified exactly two binding partners. All genetic interactions were also removed.

### Random networks

Randomised protein interaction networks with identical degree distributions were generated from the original filtered experimental interaction data for each species. In each randomisation step, a mapping is created that assigns every node a randomly chosen replacement node. In this way the edges of the network remain in place, while the nodes are shuffled randomly. It should be noted that the degree distribution per node is not maintained. Instead, this behaviour simulates a network with a high false positive rate.

### P values

P values for observations *x *were calculated as *P*(*X *≥ *x*) = *f*(*x*; *μ*, *σ*), where *f*(*x*; *μ*, *σ*) is the probability density function of the normal distribution with mean *μ *and standard deviation *σ*. *μ *and *σ *are estimated through the randomisation experiments. The density function thus provides the probability that a value less than or equal to *x *is observed by chance, given the distribution estimated by a random resampling method. Where appropriate, the inverse probability *P*(*X *> *x*) = 1 - *f*(*x*; *μ*, *σ*) was applied.

## Authors' contributions

BSB wrote all software and carried out all the analyses. AB contributed to the design and interpretation of the study.

## Supplementary Material

Additional file 1 **iPfam_pair_frequencies**. En Excel file containing the frequencies of all *i*Pfam domain pairs for all five species, i.e. the number of interactions that contain the respective *i*Pfam domain pair. Click here for file

Additional file 2 **iPfam_architectures**. Two Excel sheets containing the frequencies of all *i*Pfam domain architectures in *S. cerevisiae *and *H. sapiens *experimental interactions, i.e. the number of proteins that are composed of a certain set of *i*Pfam domains. Click here for file

Additional file 3 **iPfam_pair_conservation**. An Excel spreadsheet containing the 107 *i*Pfam domain pairs conserved amongst *E. coli*, *S. cerevisiae *and *H. sapiens *and their frequencies. Click here for file

Additional file 4 **iPfam_frequencies**. An Excel file containing the frequencies of each of the *i*Pfam domains for all five species, i.e. the number of proteins containing the respective domain. Click here for file

Additional file 5 **iPfam_inter-intrachain**. An Excel file listing the number of interchain and intrachain interactions in all PDB structures for each *i*Pfam domain pair. Click here for file
